# Crime and COVID-19: effect of changes in routine activities in Mexico City

**DOI:** 10.1186/s40163-021-00151-y

**Published:** 2021-06-30

**Authors:** Patricio R. Estévez-Soto

**Affiliations:** UCL Department of Security and Crime Science, UCL Jill Dando Institute of Security and Crime Science, 35 Tavistock Sq, London, WC1H 9EZ UK

**Keywords:** COVID-19, Crime incidence, ARIMA, Mexico City

## Abstract

**Background:**

This study aimed to determine whether crime patterns in Mexico City changed due to the COVID-19 pandemic, and to test whether any changes observed were associated with the disruption of routine activities, as measured by changes in public transport passenger numbers.

**Method:**

The first objective was assessed by comparing the observed incidence of crime after the COVID-19 pandemic was detected in the country with that expected based on ARIMA forecasts based on the pre-pandemic trends. The second objective was assessed by examining the association between crime incidence and the number of passengers on public transport using regressions with ARIMA errors.

**Results:**

Results indicated that most crime categories decreased significantly after the pandemic was detected in the country or after a national lockdown was instituted. Furthermore, the study found that some of the declines observed were associated with the reductions seen in public transport passenger numbers. However, the findings suggested that the changes in mobility explain part of the declines observed, with important variations per crime type.

**Conclusion:**

The findings contribute to the global evaluation of the effects of COVID-19 on crime and propose a robust method to explicitly test whether the changes observed are associated with changes in routine activities.

## Introduction

In an effort to counter the spread of COVID-19, governments and communities around the world have implemented local, regional and national ‘lockdowns,’ in which non-essential activities have been severely curtailed and people have been ordered to stay at home. Lockdowns are an extreme measure used to enforce social distancing, and research has shown that they have been effective in slowing the pandemic (Flaxman et al., 2020; Lau et al., 2020).

Because lockdowns have radically and drastically changed most social aspects of human life, it is widely assumed that they also have affected other social phenomena. The shift of activity from public to private places drastically affects the opportunity structures of many crime types. For example, street robbery is less likely to occur if streets are empty, shoplifting is impossible if shops are closed, and burglary involves greater risks if homes are always occupied. On the other hand, the confinement of people in a household can increase the risk of other crimes; in particular, diverse forms of domestic violence may be more likely, as victims cannot escape abusive partners.

The potential effects of pandemic-related lockdowns on crime have generated much academic interest. However, to date little research has been conducted outside of a few select countries that tend to be over-represented in criminological research: the US (Ashby, 2020b, a; Shayegh and Malpede, 2020; Campedelli et al., 2020; Mohler et al., 2020; Piquero et al., 2020), Europe (Halford et al., 2020; Buil-Gil et al., 2020; Gerell et al., 2020), and Australia (Payne et al., 2020).

Though, overall these studies have generally found decreases in crime associated with the pandemic, it is unclear if these patterns have also been seen in regions with vastly different criminogenic conditions. For example, the incidence and prevalence of crime in Latin America and the Caribbean is much higher than in the rest of the world (Muggah & Tobón, 2019), and crime phenomena occur in a context where governance is contested between non-state violent actors and the state (Müller, 2018). Furthermore, it is expected that the pandemic will wreak havoc in the economy and stability of countries in the region. The UN Economic Commission for Latin America and the Caribbean (ECLAC) estimates that the pandemic “will cause the greatest economic contraction ever,” cause unemployment to increase to 38 million, and drive 30 million people to poverty in the region (ECLAC, 2020). The combination of high crime rates, deteriorating social and economic conditions, as well as changes in illicit markets and criminal governance prompted by the crisis (Felbab-Brown, 2020; Asmann et al., 2020) beg the question of whether crime phenomena will react in the same way in Latin America as in the rest of the countries studied.

Studying the effects of the pandemic on crime in Latin America and the Caribbean is important for two main reasons. First, crime is a highly salient issue in the region and it is relevant to study if any changes observed during this period are related to the pandemic. For example, on July 28, 2020, Mexico’s security ministry claimed that they had achieved a 22% reduction in robberies against businesses during the first 6 months of the year, without acknowledging that during three of those months most businesses were closed or had gone out of businesses due to the lockdown.^1^ Second, the lockdown is the “largest criminological experiment in history” (Stickle & Felson, 2020), and thus represents a golden opportunity to test whether the theories that have been developed to explain crime in the ‘developed’ world are also suitable to explain crime in other regions.

Focusing on Mexico City, this study aims to determine if crime patterns changed due to the COVID-19 pandemic, and to test whether any changes observed were associated with the disruption of routine activities. The study proceeds as follows: In the next section I present a brief overview of the theoretical framework that motivates the study. Then I describe the data and methods used in the study, and follow with the results. This is followed by the discussion and conclusions.

### Routine activities, lockdowns and crime

Cohen & Felson, (1979) proposed the routine activity approach to explain the increase in crime in the United States after WWII—when improvements in social conditions would have predicted crime to decrease—noting that the dispersion of activities away from suburban households in the postwar period generated many crime opportunities for property crime (see also, Felson, 2017). Drawing on Hawley’s, (1950) human ecological theory, the routine activity approach did not see crimes as indicators of a malfunctioning society, but rather as a feature of everyday life.

The fundamental premise of this approach is that crime events are the product of crime opportunities, which occur when a likely offender converges with a suitable target in the absence of a capable guardian. In turn, the rate at which crime opportunities occur is a function of the routine activities that structure everyday life—e.g. work, leisure, school, family, etc. It then follows that social changes that alter routine activities can have dramatic effects on crime, even if the social factors that presumably drive criminal motivation (such as poverty and unemployment) remain stable.

While it was originally proposed to explain a crime increase, the routine activity approach has also been useful to explain the crime drop seen in many developed countries since the turn of the 21st century, as security improvements reduced target suitability for several types of predatory crime (Tilley et al., 2015; Farrell et al., 2014, 2011a, 2011b, 2010).

The most noticeable change in routine activities due to COVID-19 is a dramatic reduction in activity levels in public spaces, as people were mandated to stay at home to reduce contagion. This reduction in mobility is unlikely to affect all crime types in the same way. Crimes that take place in public (e.g. street robberies) are likely to decrease, as there are fewer likely offenders and suitable targets on the street due to the lockdown. On the other hand, crimes that take place inside households (e.g. domestic violence) may increase, as victims are less able to escape offenders living in the same household.

To date there have been a few studies of the effects of COVID-19 related lockdowns on crime. Ashby, (2020b) used ARIMA forecasting to compare the estimated incidence of crime given pre-COVID-19 trends with the counts observed in 16 US cities after COVID-19 was detected in the country. The study found no significant changes in serious assaults, reductions in burglary in some cities, little change in non-residential burglary (except in one city), decreases in thefts from vehicles, and diverging effects on thefts of vehicles. Halford et al., (2020) used a similar modeling approach to estimate the effect of COVID-19 lockdowns on crime in one UK police area, and found that all crime had declined dramatically by one week after the lockdown. Similarly, Payne et al., (2020) used ARIMA forecasting to estimate the effect of lockdowns on crime in Queensland, Australia, and found significant declines in assault and sexual assault, though domestic violence patterns were not affected. Campedelli et al., (2020) used Bayesian structural time-series models to estimate the effect of social distancing policies on crime in Los Angeles and found significant decreases in overall crime, robbery, shoplifting, theft and battery. Mohler et al., (2020) used regression analysis to estimate the difference in mean calls for service before and after COVID-19 related lockdowns in Indianapolis and Los Angeles, and found modest reductions in some crime types, though not consistently across places. In contrast, Shayegh & Malpede, (2020) used an econometric time series model to estimate the effect of lockdown orders in San Francisco and Oakland, finding a large 40% reduction in crime in both cities, though they found no reduction in domestic violence. Specifically regarding domestic violence, Piquero et al., (2020) used regressions and ARIMA forecasting to analyze the effect of lockdowns on domestic violence in Dallas. Their study found short-term increases in domestic violence in the two weeks after the lockdown, though the increases appear to predate the policy (possibly due to people reducing their mobility before official lockdown orders were issued). Gerell et al., (2020) used monthly crime projections inferred from previous years and found that total crime, assaults, pick-pocketing and burglary decreased significantly in Sweden, though reductions were somewhat modest.

All of the studies cited consider the changes in crime observed to be (at least partially) associated with the shifts in mobility following lockdowns, as predicted by the routine activity approach. However, most studies did not explicitly examine the association between changes in mobility and changes in crime, and instead used discrete before and after periods to identify changes in crime patterns. Two exceptions are Mohler et al., (2020) and Halford et al., (2020), who in addition to using discrete time periods, also examined the relationship between crime and a mobility index. Both of these studies used Google’s COVID-19 Community Mobility Reports as a measure of the change in routine activities due to COVID-19 related lockdowns (as well as capturing voluntary reductions in mobility prior to lockdowns being officially mandated). Mohler et al., (2020) used a regression model to explicitly estimate the effect on crime of a change in daily mobility, while Halford et al., (2020) used total changes in mobility observed for the study period to calculate the ‘Mobility Elasticity of Crime’ (MEC)—which captures the proportional change in crime for a one percent change in mobility.

Google’s COVID-19 Community Mobility Reports offer important advantages. Specifically they are an innovative and timely data source to measure changes in aggregated ambient population over time in six location types: retail and recreation, grocery stores and pharmacies, parks, transit stations, workplaces, and residences. However, there are important limitations to these data.^2^ First, the measures do not reflect absolute changes in mobility, but rather deviations from a baseline taken from the median value for each day of the week between Jan 3 and Feb 6, 2020. As the baseline reference values may be affected by seasonality or exceptional conditions (e.g. weather events, natural disasters, social and political protests), it is not clear how representative of “normal” mobility these baseline figures are. Second, measurements are taken from mobile phone users that use Google’s services and have opted-in to share their location. It is not clear that this subset of users is a representative sample of the ambient population at all location types, and thus it is likely that the data present some systematic measurement errors that are not yet fully understood. And third, Google’s mobility reports are only available from early 2020, thus it is not possible to examine the relationship between mobility and crime over a longer time period to determine if and how this relationship may have changed during the pandemic—a key interest in this study.

## Data and methods

The data used in this study were obtained from Mexico City’s open data portal.^3^ This portal houses 224 data sets from 29 different administrative units from Mexico City’s government. The data sets are grouped in 17 thematic categories.^4^ For this study I used data sets from the justice and security, and mobility categories.

### Justice and security data

The justice and security category houses 19 data sets. Of these, I used case files (*carpetas de investigación*) recorded by the Mexico City prosecutor’s office to estimate crime incidence in Mexico City. In contrast with common practice in other countries, the responsibility for recording crime incidents in Mexico falls on state prosecutors instead of the police.^5^ Prosecutor’s case files are created in response to a crime report submitted by a victim or a third party that has witnessed or discovered a serious crime. Thus, prosecutor’s case files exhibit similar underreporting issues as police-recorded crime, insofar they are unlikely to capture crimes not reported by victims.

Underreporting is a widespread problem in Mexico. According to Mexico’s national victimization survey (INEGI, 2020a), only 11% of all crimes experienced in the country during 2019 were reported to an appropriate authority, though only 8% of all crimes led to a case file in the state prosecutor’s office. Reporting rates are slightly worse in Mexico City, where only 8% of all crimes were reported and only 6% of all crimes led to a prosecutor’s case file (INEGI, 2020b). National figures suggest that there are important variations in the reporting rate by crime type: the most widely reported crime type is car theft (61% of incidents were reported), with most other crime types exhibiting reporting rates between 18% and 3% (INEGI, 2020a). However, the reporting rate has remained relatively stable over the past decade, which suggests that changes in case file trends are reasonably able to capture changes in crime incidence.

Despite low reporting rates, prosecutor’s case files are routinely used for policy purposes, as well as for scholarly research (e.g. Pérez Morales et al., 2015; Vilalta et al., 2021, 2020; Vilalta et al., 2018; Denegri & Ley-García 2021; Echarri Cánovas, 2012). In contrast with Mexican victimization surveys—which are updated yearly and are aggregated at the city level—estimates of crime incidence from case files are updated monthly and are published at the incident level in Mexico City. While it is widely acknowledged that they only capture a subset of the true incidence of crime, case file statistics are the only available measure to track the evolution of crime in monthly, weekly or daily time series.

To prepare the data sets for analysis, I downloaded all case files for crimes recorded in Mexico City from Jan 1, 2017 to May 31, 2020. Case files were classified into six broad crime categories—as well as an “all crimes” category—and aggregated by date of occurrence to construct 7 time series of daily crime counts covering the period from Jan. 1, 2017 to May 24, 2020.^6^ The crime categories used were:

- All crimes.
- Violent robbery,
- Non-violent robbery.
- Robbery against residence.^7^
- Serious violent crime (non-sexual).^8^
- Sexual violence.^9^
- Domestic violence.

The categories were chosen in an attempt to capture how the changes in routine activities prompted by the COVID-19 pandemic could affect the different opportunity structures underpinning various crime types. In particular, it was hypothesized that incidents that tend to disproportionally affect women more than men (i.e. sexual and domestic violence) were likely to increase as people were confined in their homes (and thus victimized women were more likely to be trapped with their abusers).

### Violence against women

As sexual and domestic violence tend to suffer from particularly high under-reporting rates, I used a data set with the number of calls to a women’s helpline to triangulate the amount of crimes involving violence against women. Though the helpline advises women on a range of issues, I selected only those calls that were related to gender-related violence^10^ and constructed time series with daily counts of calls from Jan. 1, 2017 to May 24, 2020.

### Mobility data

Lastly, to estimate the changes in routine activities associated with the pandemic, I used figures on the daily number of passengers on two of Mexico City’s largest public transport systems: the Metrobus bus rapid transit (BRT), and the Metro (SCT). Though there are additional public transport systems in the city (including several light rail, bus, tram and bike sharing systems), passenger data for the entire period studied was only available for BRT and SCT. After excluding passenger numbers for lines opened after Jan. 1, 2017, passenger numbers from both services were combined into a daily time series.

Public transport passenger numbers have important advantages over Google’s COVID-19 Community Mobility Reports. First, public transport data are available in absolute figures, rather than as deviations from an arbitrary baseline. Thus, it is possible to control for seasonality and other exceptional conditions that may have affected passenger numbers in a particular day. Second, public transport represents a large share of mobility in Mexico City: it is estimated that around 50% of all trips taken in Mexico City in 2017 used public transport as the main means of transportation (INEGI, 2018).^11^ And third, there are more than 10 years of publicly available daily public transport passenger figures, which allows estimating the relationship between crime and mobility for the period before the pandemic.

A disadvantage of public transport passenger numbers is that they cannot capture how ambient population changed in a variety of location types as captured by Google’s COVID-19 Community Mobility Reports. However, given that around 1 in every 2 trips in Mexico City use public transport, passenger numbers are taken as a proxy measure for overall mobility in the city, with the assumption being that large reductions in public transport passenger numbers during the pandemic are indicative of the large scale disruption to the city’s routine activities.

### ARIMA forecasting

As Ashby, (2020b) notes, determining the effect of the COVID-19 pandemic on crime requires comparing the observed incidence with that expected in the absence of the pandemic. However, because the pandemic is by definition a global event, there are no suitable locations that could be used as controls as is common in natural experiments. Thus, to estimate a suitable counterfactual expectation, I used autoregressive integrated moving average (ARIMA) forecasting models with seasonal components. Time series forecast models are based on the fundamental notion that past observations of a phenomenon are generally good predictors of future observations (Box-Steffensmeier et al., 2014). Because the past tends to affect the future in complex (sometimes simultaneous) short, medium and long term dependency processes (Box-Steffensmeier et al., 2014), ARIMA models seek to capture this complexity by specifying the number of non-seasonal and seasonal autoregressive, integration, and moving average parameters—denoted as $ARIMA(p,d,q)\times (P,D,Q)_{period}$—required to reduce the time series to a white noise process.

In this study, and following Ashby, (2020b) and Halford et al., (2020), ARIMA parameters were automatically selected using the algorithm outlined by Hyndman & Khandakar, (2008), which iteratively selects the parameters that minimize the corrected Akaike information criterion (AICc),^12^ as implemented by the ‘fable’ package (O’Hara-Wild et al., 2020) in R (R Core Development Team, 2015).

ARIMA models were trained using the period before the pandemic (Jan. 1, 2017 to Feb. 28, 2020). Two models per variable were estimated: a) one with a stochastic trend (i.e. in which the trend is allowed to vary over time) or b) one including a deterministic trend (i.e. in which the trend is assumed to be a constant function of time) (see Box-Steffensmeier et al., 2014, p. 23–26). For most variables, models with a stochastic trend had better in-sample accuracy measures than models with deterministic trends. The only exceptions were sexual and domestic violence, and VAW helpline calls, in which a deterministic trend offered a better fit (see Table 2 for the final specifications used).

Because the variables used are strictly positive counts (i.e. with a lower bound of 0), the dependent variable was log-transformed, ensuring that predictions would not be negative counts (see Gelman & Hill, 2007, p. 59).

The models were then used to forecast the values and 95% confidence intervals expected for all days between Feb. 29 to May 24, 2020. To summarize the effect of the pandemic, I calculated the percentage change between the observed and forecast values. To calculate confidence intervals for these estimated changes, I also calculated the percentage change between the observed value and the upper and lower bounds of the forecast confidence interval.

### Crime-mobility models

A problem with previous studies that have examined the relationship between mobility and crime during the pandemic (i.e. Halford et al., 2020; Mohler et al., 2020) is that the methods used to quantify the relationship did not take into account the time series structure of the data. Estimating the relationship between two longitudinal variables without adequately accounting for their time series properties can easily lead to a ‘spurious regression’, where it is falsely concluded that there are significant relationships between unrelated non-stationary variables  (Box-Steffensmeier et al., 2014, p. 125-126). Furthermore, the presence of autocorrelation in the error terms can bias inferences. Thus, to estimate the relationship between crime and mobility, I used linear models with non-seasonal and seasonal ARIMA errors (Hyndman, 2010; Hyndman & Athanasopoulos, 2019). The general form of the models used (in backshift notation) is:

$$\begin{aligned} log(y_t) = \beta log(x_t) + \frac{\theta (B)\Theta (B^7)}{\phi (B)\Phi (B^7)}z_t \end{aligned}$$

where $y_t$ and $x_t$ are the time series of the dependent and independent variables, $\beta$ is the coefficient estimate quantifying the relationship between $y_t$ and $x_t$, $\phi$ and $\theta$ are the non-seasonal autoregressive and moving average terms, while, $\Phi$ and $\Theta$ are the autoregressive and moving average terms for weekly seasonal effects, *B* is the backshift operator, and $z_t$ are the white noise residuals. If differencing is required to control for (non-seasonal) integration,^13^$\phi (B)$ is replaced by $\nabla ^d \phi (B)$, where $\nabla$ is the differencing operator ($1-B$) (Hyndman, 2010; Hyndman & Athanasopoulos 2019). It is relevant to note that this differencing procedure is equivalent to fitting a model with ARMA errors to differenced time series of $y_t$ and $x_t$ (Hyndman, 2010).

Selection of the number of ARIMA error terms followed the same automatic approach as that outlined in the previous section. Similarly, I also tested whether deterministic trends were required. After an initial estimation, residuals were checked for autocorrelation using the Ljung-Box test (Ljung & Box, 1978) at 21 lags, and for stationarity using the KPSS test (Kwiatkowski et al., 1992). These tests revealed that the automatically selected ARIMA parameters for all crimes, violent robbery, serious violent crime, domestic violence, and VAW helpline calls were inadequate, as the residuals were autocorrelated and there were indications of non-stationarity. Thus, I fitted further ARIMA models manually selecting parameters for these crime types until the residuals resembled a white noise process.

As the dependent and independent variables are log-transformed, the mobility coefficient represents approximately the percentage change expected in *y* for a 1% change in *x*, and thus can also be interpreted as the elasticity (Wooldridge, 2013, p. 44). In this sense, the mobility coefficient can be viewed as a more reliable version of the Mobility Elasticity of Crime (MEC) reported by Halford et al., (2020), insofar as it controls for the time series properties of *x* and *y*, whereas the MEC does not.

Interpretation of the mobility coefficient is as follows: if the coefficient were 1.5, it would mean that crimes increase (decrease) 1.5% for a 1% increase (decrease) in mobility.^14^ To estimate the effect for a different magnitude (for example for a 50% reduction in mobility), the following expression can be used: $0.5^{\beta }$. Thus, for a hypothetical mobility coefficient of 1.5, crimes would be expected to decrease by 55% for a 50% reduction in mobility ($0.5^{1.5} = 0.45$).

The crime-mobility models with ARIMA errors were fitted to the entire observation period (Jan. 1, 2017 to May 24, 2020). However, as I was also interested in examining whether the relationship between mobility and crime changed due to the pandemic, I estimated additional models using two data subsets. One examined the period before COVID-19 was detected in the country, to estimate the ‘normal’ relationship between mobility and crime; and the other was fitted to the period after the lockdown, to capture the relationship between mobility and crime during the pandemic. Observations after COVID-19 was detected and before the lockdown began were excluded from these secondary analyses to avoid potential contamination effects.

## Results

**Fig. 1:**
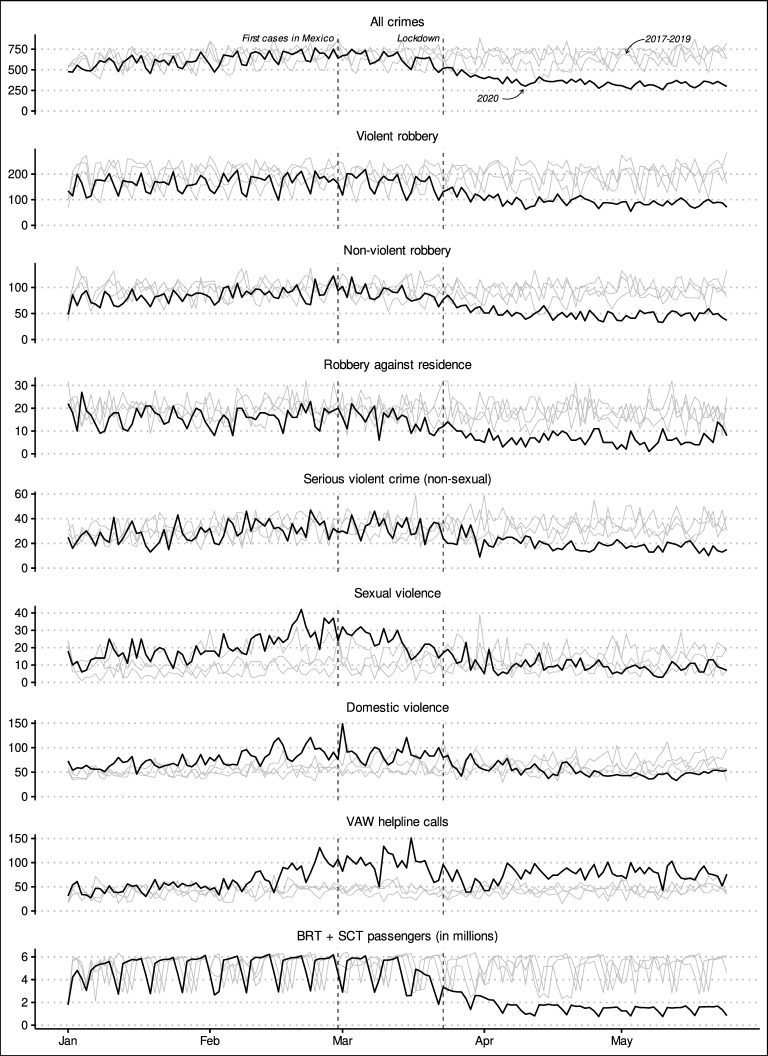
Daily counts of the variables of interest from Jan. 1 to May 24, 2020, compared with the counts observed between 2017 to 2019

Before presenting the results of the ARIMA forecasting and crime-mobility models, I briefly present descriptive statistics of the time series analysed. Table 1 presents descriptive statistics for the period before COVID-19 was detected in the country (Jan. 1, 2017 to Feb. 28, 2020), after COVID-19 was detected (Feb. 29, to May 24, 2020), as well as for the entire period. Figure 1 compares the time series for each variable from Jan. 1 to May 24, 2020, with the observations for the same period in 2017 to 2019. The plots also feature dashed lines indicating two significant dates: the day the first COVID-19 cases were reported in the country (Feb. 29, 2020), and the day the national mandated lockdown began (Mar. 23, 2020).

**Table 1:** Descriptive statistics for the variables of interest

	Pre–COVID–19		Post–COVID–19			Pre– and post–COVID–19	
	Mean (sd)	Range	Mean (sd)	Range	% change	Mean (sd)	Range
All crimes	654.3 (91.9)	[332–942]	433.0 (139.7)	[259–748]	−33.8%	638.9 (111.2)	[259–942]
Violent robbery	195.9 (42.8)	[50–309]	113.8 (39.7)	[54–218]	−41.9%	190.3 (47.4)	[50–309]
Non-violent robbery	93.4 (17.1)	[32–140]	60.4 (20.3)	[33–120]	−35.3%	91.1 (19.3)	[32–140]
Robbery against residence	19.0 (5.0)	[5–36]	9.0 (4.9)	[1–22]	−52.6%	18.3 (5.6)	[1–36]
Serious violent crime (non-sexual)	32.0 (7.6)	[13–70]	22.7 (8.3)	[9–46]	−29.1%	31.4 (8.0)	[9–70]
Sexual violence	12.6 (6.6)	[1–42]	13.4 (7.8)	[3–32]	6.3%	12.7 (6.7)	[1–42]
Domestic violence	59.8 (15.5)	[22–122]	62.4 (21.3)	[33–149]	4.3%	59.9 (16.0)	[22–149]
VAW helpline calls	44.1 (13.0)	[8–131]	81.5 (20.4)	[39–151]	84.8%	46.7 (16.6)	[8–151]
BRT + SCT passengers (in millions)	5.2 (1.1)	[1.8–6.6]	2.4 (1.5)	[0.8–6.1]	−53.8%	5.0 (1.3)	[0.8–6.6]

These exploratory analyses suggest that all crimes, both robbery types, and serious violent crimes experienced marked declines after the pandemic began in Mexico. As Table 1 indicates, these crimes decreased by between 29.1% and 52.6%. In contrast, sexual and domestic violence increased by a small amount, and VAW helpline calls appear to have jumped by 84.8%. The time series in Fig. 1 for all crimes, robberies, and serious violent crimes suggest that the declines for these crimes were associated with the pandemic, as the downward trends begin about two weeks after the first cases were detected in the country and they stabilise at a low level about 3 weeks after the lockdown. In contrast, the time series for sexual and domestic violence, and for VAW helpline calls suggest that these crimes were trending upward before the effects of the pandemic would be expected (late January and early February). Sexual and domestic violence peaked in the last weeks of February and in early March, remained at an elevated level through March, and declined after the lockdown (though they remained at roughly the same levels seen in 2017–2019). VAW helpline calls peaked in late February, remained at an elevated level the first half of March, and experienced two further peaks in the second half of March. Calls then dipped following the lockdown for about two weeks, and in April they rose to the level seen in late February (a level higher than that seen in previous years). Lastly, Table 1 and Fig. 1 also show how daily passenger numbers changed in response to the pandemic: the mean daily number of passengers using Mexico City’s BRT and SCT systems decreased by 53.8% compared with the period from Jan. 1, 2017 to Feb. 28, 2020. The patterns suggest that the decline in urban mobility began about a week before the official lockdown was mandated and stabilised below 2 million passengers per day from mid April onward.

In any case, these exploratory analyses do not take into account the complex time series patterns present in the data which require more sophisticated modeling approaches, the results of which are presented in the following sections.

### ARIMA forecasting

**Fig. 2:**
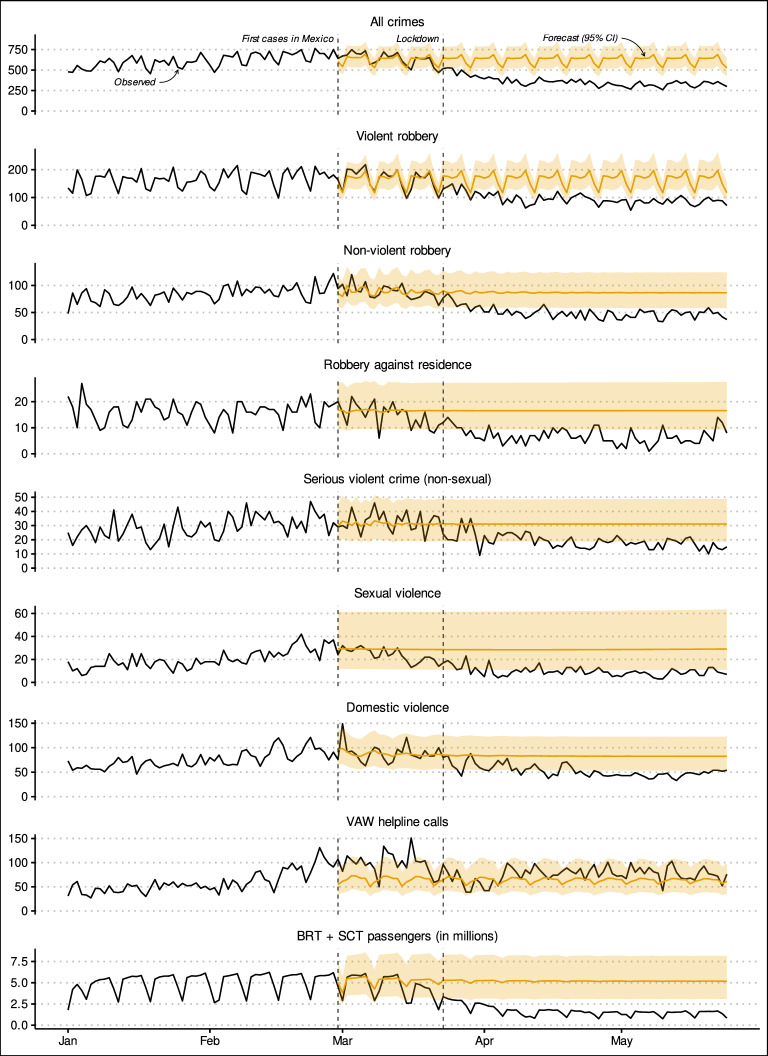
Daily counts of crimes and public transport passenger numbers from Jan. 1 to May 24, 2020 compared to counterfactual estimates based on ARIMA models estimated using data from Jan. 1, 2017 to Feb. 28, 2020

The specifications and accuracy measures of the ARIMA forecasting models are shown in Table 2 (see Appendix 1), while the forecasts and confidence intervals are shown in Fig. 2. Looking at the results shown in Table 2 first, the (in-sample) accuracy measures for the pre-COVID-19 period suggest that the ARIMA models captured the complexity of the time series to a reasonable extent. In contrast, the (out-of-sample) accuracy measures for the post-COVID-19 periods provide a first estimate of the effect of the pandemic: The mean error for all variables (except VAW helpline calls) is negative, indicating that the observed values were consistently smaller than those expected before the pandemic.

While the overall patterns shown in Figure 2 broadly agree with those shown in the exploratory analysis presented earlier (see Figure 1), the 95% confidence interval permits making a more robust comparison to the counterfactual. The aggregated “all crimes” variable began gradually declining about two weeks after the first COVID-19 cases were detected in Mexico, though the decline was only significantly different from the forecast from about 3 days after the lockdown began and remained at a significantly low level for the duration of the study period. Violent robbery similarly declined significantly for the majority of the study period, though the decline began about a week later—after the lockdown was instituted—and was statistically significant from early April onward. In contrast, the decline in non-violent robbery appears to have started about a week before the lockdown, and was similarly significant from early April throughout the rest of the period, except for one day. Robbery against residence followed a very similar pattern, though there were more days in which the decline was not significant, and the last days of the period suggested a slight upward trend. In contrast with most other crimes, serious violent crimes declined more abruptly in early April after remaining fairly stable during March. Furthermore, while serious violent crimes remained at a low level throughout the rest of the period, many observations were not significantly lower than the expected incidence.

Sexual violence appeared to have declined gradually from about a week before the lockdown and stabilized at a low level from early April onward. While many observations were significantly lower than expected, the observations were generally close to the lower bound of the expected threshold. Domestic violence had a significant peak right after the first cases of COVID-19 were detected in the country, however, due to its timing it is unlikely to be causally related to the pandemic. The incidence of domestic violence remained at the expected level through March and began declining after the lockdown, though the declines were only consistently significant until late April onward. The forecast for VAW helpline calls suggests that the peaks observed before COVID-19 cases were detected in the country, and which continued through March, were anomalous and unlikely to be related to the pandemic. The incidence of VAW calls appears to have dropped to the expected levels after the lockdown, dip for about a week in late March and early April, and increase to a level higher than that expected afterwards—though observations were consistently within the expected forecast intervals.

The final plot in Figure 2 shows the effect of the pandemic on urban mobility. The observed number of passengers using public transport shows a gradual decline about two weeks after the first cases of COVID-19 were detected in the country when compared with the forecast. The decline became statistically significant around the date of the lockdown and remained at a stable and statistically significant low level for the remainder of the study period.

**Fig. 3:**
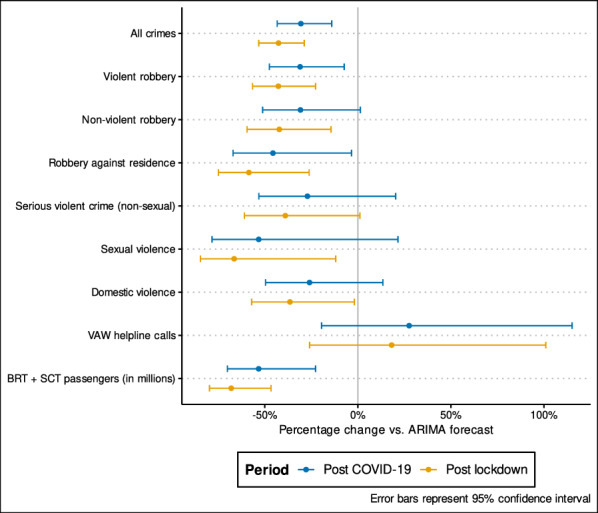
Percentage reduction in total crime and public transport passenger numbers observed between Feb. 29 to May 24, 2020, compared to counterfactual estimates based on ARIMA forecasting models

Figure 3 presents summaries of the total impact of the pandemic (a table with point estimates can be found in the Appendix 1) for two periods: The ‘post-COVID-19’ period from Feb. 29 to May 24, 2020; and the post lockdown period from Mar, 23 to May 24, 2020. All crimes declined by 30.6% during the post-COVID-10 period, and by 42.7% after the lockdown, and as the confidence interval did not cross 0%, the declines were statistically significant. Similarly, violent robberies declined significantly by 31.0% and 42.7% during the two periods. The overall decline for non-violent robbery during the entire post-COVID-19 period was 30.9%, though the confidence interval suggests that this decline was not significant. However, if we only consider the post lockdown period, the 42.2% decline was statistically significant. Robbery against residence decreased significantly by 45.6% and 58.6% during the post-COVID-19 and post lockdown periods, respectively. While the total incidence of serious violent crimes decreased by 27.1% and 39.0% during both periods, the declines were not statistically significant, as the confidence intervals cross 0%.

Sexual violence declined by 53.3% during the post-COVID-19 period (though it was not statistically significant), and by a statistically significant 66.5% after the lockdown. Similarly, domestic violence was down by a non-significant 26.0% during the entire post-COVID-19 period, and by a (barely) significant 36.5% after the lockdown. In contrast with all other crimes, total VAW helpline calls increased by 27.6% and 18.15% over the expected during the two periods, though these increases were not statistically significant.

Lastly, the number of passengers using the BRT and SCT public transport systems decreased by 53.3% and 68.1% during the post-COVID-19 and post-lockdown periods, respectively. Both estimates were found to be statistically significant.

### Crime-mobility models

The main results of the crime-mobility models with ARIMA errors are shown in Table 3 (see Appendix 1). Overall, all models were reasonably accurate and achieved a good fit. Ljung-Box Q statistics suggested autocorrelation was not significant overall for most variables. The only exception was VAW helpline calls, where the Q statistic indicated (marginally) significant autocorrelation ($p=0.048$), despite attempts to eliminate it trying different ARIMA specifications.^15^ This means that, for VAW helpline calls, the standard error for the mobility coefficient may be somewhat biased—though the coefficient is correctly estimated—thus interpretation should be conducted with caution. On the other hand, the errors for all variables showed no signs of non-stationarity as all KPSS test statistics were not significant.

In general, mobility coefficients were significantly and positively associated with crime incidence, with the exception of robbery against residence and domestic violence, as their mobility coefficients were not statistically significant. Nonetheless, the association between mobility and crime was inelastic. A 1% increase in mobility was associated with a 0.16% increase in all crimes; a 0.30% and 0.37% increase in violent and non-violent robbery, respectively; an essentially negligible 0.08% increase in non-sexual violent crime; a 0.22% increase in sexual violence; and a 0.14% increase in VAW helpline calls.

Though these effects appear small, they can be better appreciated by calculating the predicted change expected given the dramatic reduction in mobility observed during the pandemic. Considering only significant coefficients, the 68.1% reduction in mobility estimated for the post-lockdown period, predicts a 17.3% reduction in all crimes. Similarly, violent and non-violent robberies would decline 29.4% and 34.7%. Serious violent crimes would decrease 8.8%, sexual violence 22.4%, and VAW helpline calls 14.4%.

**Fig. 4:**
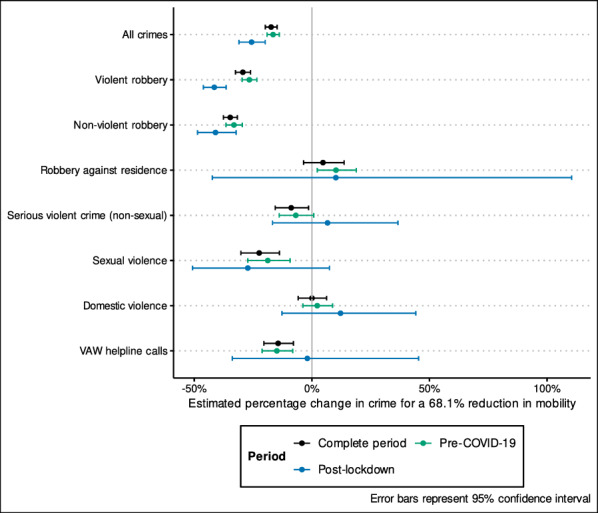
Estimated percentage change in daily crime rates for a 68.1% reduction in daily passenger numbers, using three time periods

The secondary analyses examining the pre-COVID-19 and post-lockdown periods suggested that the relationship between mobility and crime was generally consistent during the pandemic, with some variation in strength and significance per crime type. The differences in the mobility coefficient for the three periods studied (complete period, pre-COVID-19 and post-lockdown) are summarised in Fig. 4, which presents the predicted change in crime for a 68.1% reduction in mobility (point estimates can be found in the Appendix 1). Effect sizes and standard errors for the pre-COVID-19 period were generally consistent with those from the complete period. In contrast, effect sizes showed more variability and standard errors were consistently greater for the post-lockdown estimates.^16^

The coefficients for all crimes, and violent and non-violent robbery were consistent insofar as the models predicted statistically significant declines during the three periods; however, the post-lockdown estimates suggest that the effect of mobility on crime was greater during the pandemic. During the pre-COVID-19 period, a 68.1% reduction in mobility was associated with a 16.5% decrease in all crimes, while during the post-lockdown period the expected decrease was 25.6%. For violent robbery, the pre-COVID-19 relationship predicted a 26.6% decrease for a 68.1% reduction in mobility, while the post-lockdown period estimate suggested a 41.5% decrease. Similarly, for non-violent robbery, the pre-COVID-19 relationship was predicted a 33.1% crime reduction for a 68.1% drop in mobility, while the post-lockdown estimate predicted a 41.0% reduction in non-violent robbery. The mobility coefficient for robbery against residence was inconsistent across the two periods. While, the estimates from the pre-COVID-19 and post-lockdown periods suggested an increase of 10.3% in robberies against residences would be expected for a 68.1% reduction in mobility, this was only statistically significant for the pre-COVID-19 period.

Though the complete period model had suggested that there was a small, positive and statistically significant relationship between mobility and serious violent crime, the coefficients from the pre-COVID-19 and post-lockdown models were not significant, suggesting that the relationship is unlikely to be significant. The coefficients for sexual violence were consistent in terms of direction and magnitude, with the pre-COVID-19 relationship predicting an 18.7% decline in sexual crimes for a 68.1% reduction in mobility; however, the estimates from the post-lockdown period were not significant. As in the complete period model, the mobility coefficients for domestic violence were not statistically significant in the pre-COVID-19 and post-lockdown periods. Lastly, the mobility estimates for VAW helpline calls from the pre-COVID-19 period predicted a statistically significant decline of 14.9% calls for a 68.1% reduction in mobility (similar to the complete period estimate). However, the estimate from the post-lockdown period was not statistically significant.

## Discussion

This study sought to investigate whether crime patterns in Mexico City changed due to the COVID-19 pandemic, and to test whether any crime changes observed were associated with the disruption of routine activities as captured by changes in public transport passenger numbers. Two analyses were carried out. First, the observed incidence of crime after the COVID-19 was detected in the country was compared with ARIMA forecasts based on the pre-pandemic trends. Then, the relationship between crime incidence and public transport passenger numbers was examined using regressions with ARIMA errors. Overall, the findings suggested that crime declined markedly after the COVID-19 was detected in the country, and that part of these decreases were associated with the change in routine activities. However there were important variations in magnitude and significance per crime type.

An aggregated measure of all crimes saw statistically significant reductions after the pandemic, and the mobility coefficient was significant and positive (suggesting all crimes decreased in line with urban mobility). This suggests that at least part of the reduction observed in all crimes was likely associated to the change in routine activities captured by the reduction in mobility. However, it is relevant to note the strength of the association between all crimes and mobility appeared to increase after the COVID-19 lockdown was mandated in the country, which suggests the presence of an interaction between mobility and an unobserved variable related to the lockdown. For example, in addition to affecting the supply of suitable targets, reductions in mobility could have made reporting crimes more difficult.

Similarly, violent and non-violent robberies experienced significant reductions during the pandemic (though the reduction in non-violent robberies was only significant for the period after the lockdown), and the mobility coefficient suggested that part of these declines can be explained by the reductions in public transport mobility. Like for all crimes, the association between mobility and violent and non-violent robberies appeared to strengthen in the post-lockdown period—similarly suggesting the presence of an interaction.

Though robberies against residences experienced a significant decline during the pandemic, the mobility coefficient was not significant, which suggests that the decline observed in this crime type is unlikely to be explained by the change in mobility patterns and may instead be driven by other changes in routine activities associated with the pandemic.

Serious violent crimes experienced non-significant reductions during both post-COVID-19 periods. This was consistent with the mobility coefficient which did not suggest a relationship between urban mobility and serious violent crime.

I had hypothesized that crimes that disproportionately affect women would increase during the pandemic as women would be less able to escape their potential abusers (and hence would have a negative relationship with urban mobility). For sexual violence this expectation was informed by global patterns suggesting that the majority of sexual violence occurs in homes rather than in public places (Jewkes et al., 2002, p. 161). Domestic violence, by definition, is expected to occur mostly in homes. However, contrary to expectations, sexual and domestic violence crimes decreased during the pandemic (though this was only significant for the post-lockdown period). The results of the crime-mobility models help clarify these findings.

The mobility coefficient for sexual violence crimes was significant and positive, suggesting that as mobility decreased, the incidence of sexual crimes decreased as well. This surprising finding may be explained by the fact that sexual assault is extremely common in Mexico City’s public transport: 80% of female users reported being a victim of sexual violence on public transportation in the past year (Zermeño Núñez & Plácido Ríos, 2009). Thus, it is conceivable that the decrease in sexual assaults during the pandemic could be the result of fewer women using public transport. However, while the mobility coefficient for the post-lockdown period was of similar magnitude than for the pre-COVID-19 period, the estimate was not significant, thus it is not possible to establish this with certainty. In any case, this would appear to be a relevant area for future research.

In contrast, the mobility coefficient for domestic violence was not significant, which suggests that the decline observed for this crime type cannot be explained by the reduction in mobility. As mentioned earlier, a potential explanation for the declines may be associated with difficulties in reporting crime, rather than true reductions in incidence.

As stated in an earlier section, I used calls to a violence against women helpline to triangulate the amount of crimes involving violence against women, as crimes of this nature tend to be severely underreported. While VAW helpline calls increased during the post-COVID-19 and post-lockdown periods (though not significantly), the mobility coefficient was only significant in the complete and pre-COVID-19 periods, and not in the post-lockdown period. Thus, it is not possible to establish whether the increases observed during the pandemic were associated with the reductions in mobility.

On the other hand, it is relevant to remember that the exploratory analyses had indicated that increases early in the pandemic in sexual and domestic violence, and VAW calls were unlikely to be causally associated with the pandemic, as they predate the first cases of COVID-19 in the country. According to González Schont, (2020), the increase in VAW calls (and presumably in sexual and domestic violence crime reports as well) before the pandemic could be explained by the fact that it coincided with events related to International Women’s Day (March 8th), which in 2020 included enhanced publicity campaigns to increase awareness of domestic violence and promote crime reporting, as well as unprecedented massive protests staged by women in response to government inaction to counter rising levels of femicides and violence against women (Averbuch, 2020; Phillips, 2020). Thus, it is possible that the increases reflect women’s heightened awareness of the VAW helpline and more willingness to report abuse, or they could also reflect true increases in the incidence of violence against women associated with a backlash effect, as a deeply patriarchal society responded violently to the feminist movement (González Schont, 2020). In any case, the unique circumstances affecting the pre-pandemic trends for sexual and domestic violence, and VAW calls threaten the internal validity of the findings for these variables, as they can affect the validity of the expected counterfactuals.

The crime reductions predicted by the observed drop in mobility during the pandemic tended to be smaller or not consistent with the drops in crime estimated using ARIMA forecasts. For all crimes, the reduction predicted by the estimated 68.1% post-lockdown drop in mobility explained only 60% of the crime drop estimated using the ARIMA forecast for the same period. For robberies against residences, and sexual and domestic violence, the mobility coefficient did not predict a statistically significant crime reduction, while the ARIMA forecasts did suggest a crime decline during the pandemic. These discrepancies suggest that, for some crime types, the changes in routine activities (as estimated by reductions in public transport mobility) may only be responsible for a fraction of the reductions observed, if at all. Two noteworthy exceptions are violent and non-violent robberies, as the mobility-predicted reductions for these crimes explained around 97% of the reductions estimated using the ARIMA forecasts for both crime types. This suggests that the declines in violent and non-violent robbery experienced after the lockdown can be largely explained by the reductions in mobility.

One possibility to explain the discrepancies is the presence of an unobserved variable associated with the onset of the pandemic. For example, as mentioned earlier, the willingness (or ability) to report a crime may have decreased during the pandemic, thus part of the reductions not explained by changes in mobility may reflect an increase in under-reporting, rather than changes in the ‘true’ incidence of criminal phenomena. As discussed in an earlier section, crime data in Mexico are collected and published by state prosecutors and experience high rates of under-reporting. While Mexico City’s government made important efforts to facilitate crime reporting and kept prosecutor’s offices working with reduced staff during the lockdown, it is likely that some victims may have avoided reporting crimes due to fear of contagion or faced other lockdown-associated hurdles.

This raises the issue of whether prosecutors case files are suitable to capture the impact of COVID-19 on crime incidence. While under-reporting rates have remained stable over the past decade, the unprecedented disruption caused by the pandemic is likely to have affected reporting behaviour. However, as mentioned earlier, case files are routinely used for policy evaluation and crime research in Mexico, as no other comparable crime data exists. Nonetheless, as data that are less affected under-reporting (such as victimization surveys) become available, future studies should reexamine the impact that the COVID-19 pandemic may have had on crime in Mexico City.

There are, of course, other limitations. In terms of internal validity, the main limitation is the absence of a suitable control to rule out competing explanations for the declines in crime incidence attributed to the pandemic. In particular, the federal and Mexico City governments have been quick to suggest that the declines in crime observed in 2020 were the result of security policies implemented after new administrations took power in late 2018. There is some anecdotal evidence to back this claim, as some crimes—such as homicide, kidnapping and car theft—experienced moderate declines before the pandemic. However, there are at least three reasons to be skeptical. First, some evidence suggests that the crime declines that the governments attribute to their policies actually began before the new administrations were sworn in (e.g. Garrido, 2019). Second, there is no rigorous evidence indicating whether any of the policies implemented by the newly-elected federal or Mexico City governments have been, in fact, effective in reducing crime. And third, even if new policies implemented since late 2018 did contribute to crime reductions in Mexico City, it is not clear why their effects would suddenly materialise more than a year later, precisely at the same time as the COVID-19 lockdown began. Thus, despite the lack of a research design that can unambiguously reject rival explanations, it is unlikely that the crime declines reported in this study were significantly driven by government policies.

In terms of external validity, an important limitation to this study is that the findings reported herein are only valid for the city and period under study. Though evidence of the impact of the COVID-19 pandemic on crime is rapidly accumulating—pointing to sizable reductions in crime due to a reduction of offending opportunities—the specific estimates presented here cannot be generalised to other cities in Mexico or the region. The task of estimating the effect of the pandemic on crime, especially in the non-Western world, remains an important avenue for future research. Similarly, the effects reported here are only valid for the period under study. After this study was completed, Mexico City partially eased lockdown restrictions by the end of June 2020, and entered a second lockdown in late December, 2020 until mid-February, 2021. While it is not yet clear how the changing restrictions may have affected mobility and crime in the city in these subsequent periods, it is likely that some of the patterns reported here have continued to a degree, given that the pandemic has continued to ravage the city with particular ferocity—there have been an estimated 91,664 excess deaths to March 15, 2021 in Mexico City, amounting to around 1% of the city’s population.^17^

## Conclusions

This study sought to determine if crime patterns in Mexico City changed due to the COVID-19 pandemic, and to test whether any changes observed were associated with the disruption of routine activities. The first objective was achieved by comparing the observed patterns for seven crime types, as well as an aggregated all crimes category, with those expected using ARIMA forecasts. The second objective was achieved by examining the relationship between the different crime categories and the number of public transport passengers, using regressions with ARIMA errors.

The analyses were consistent with findings reported in other countries, insofar they suggested that most crime categories decreased during the pandemic. Decreases were not statistically significant for serious violent crimes. While sexual violence and domestic violence exhibited significant declines after the lockdown was instituted, calls to a violence against women helpline did not reveal significant changes during the pandemic.

Furthermore, the study found that some of the declines observed were associated with the changes in routine activities as measured by the number of passengers using public transport. However, the declines predicted by the changes in mobility were not as large as—nor always consistent with—those observed, which suggested that additional processes could have affected crime incidence during the pandemic. In particular, the role of changes in reporting behaviour was discussed.

The study contributes to the global evaluation of the effects of COVID-19 on crime rates in two ways. First, it focuses on a different context outside of the countries where criminological research has traditionally been carried out. And second, it proposes a robust and explicit approach to examine the association between changes in crime with changes in routine activities in the context of the pandemic.

## Data Availability

Data and code used to generate the analyses presented in the article are available in the project repository: https://doi.org/10.17605/OSF.IO/4K9DS.

## Appendix 1

See Tables 2, 3, 4, 5.

**Table 2:** ARIMA forecasting models specifications and accuracy measures

	Specification	Pre–COVID–19		Post–COVID–19	
		ME	MAPE	ME	MAPE
All crimes	ARIMA (3,0,0)(0,1,1) [7]	3.75	6.30	−191.27	59.08
Violent robbery	ARIMA (1,0,1)(2,1,1) [7]	1.42	9.11	−51.25	60.35
Non-violent robbery	ARIMA (1,1,1)(2,0,0) [7]	1.18	12.19	−26.96	62.04
Robbery against residence	ARIMA (0,1,1)(2,0,0) [7]	0.56	22.33	−7.56	163.38
Serious violent crime (non-sexual)	ARIMA (2,1,1)(2,0,0) [7]	0.85	18.30	−8.44	60.15
Sexual violence	ARIMA (1,0,1) w/linear trend	0.82	35.83	−15.29	196.12
Domestic violence	ARIMA (2,0,1)(2,0,0) [7] w/linear trend	1.01	14.77	−21.90	51.03
VAW helpline calls	ARIMA (0,0,2)(1,0,1) [7] w/linear trend	1.12	18.08	17.60	24.36
BRT + SCT passengers (in millions)	ARIMA (4,0,0)(2,0,0) [7] w/mean	0.10	16.39	−2.77	191.91

ME is the mean forecast error, MAPE is the mean absolute percentage error

**Table 3:** Crime-mobility models with ARIMA errors: estimates, specifications and GOF measures

	Mobility coefficient (SE)	Specification	ME	MAPE	AICc	Q (df)	KPSS
All crimes	0.167 (0.014)***	ARIMA (1,1,1) (1,0,1) [7]	0.73	6.28	−2588.28	20.33 (15)	0.09
Violent robbery	0.304 (0.020)***	ARIMA (1,1,2) (1,0,1) [7]	0.18	8.81	−1774.99	16.81 (14)	0.13
Non-violent robbery	0.373 (0.020)***	ARIMA (0,1,2) (2,0,0) [7]	0.83	11.26	−1256.83	18.30 (15)	0.42
Robbery against residence	-0.040 (0.037)	ARIMA (0,1,1) (2,0,0) [7]	0.48	23.88	443.62	15.04 (16)	0.29
Serious violent crime (non-sexual)	0.080 (0.035)*	ARIMA (1,1,1) (1,0,1) [7]	0.50	17.98	−210.57	21.96 (15)	0.15
Sexual violence	0.222 (0.047)***	ARIMA (1,0,1) w/linear trend	0.83	35.60	1342.43	21.79 (16)	0.17
Domestic violence	0.000 (0.027)	ARIMA (1,1,1) (1,0,1) [7] w/linear trend	0.52	13.61	−824.66	22.19 (14)	0.10
VAW helpline calls	0.136 (0.033)***	ARIMA (1,1,1) (1,0,3) [7]	1.02	17.26	−245.83	22.52 (13)*	0.07

Notes: Mobility coefficients in log-scale; $$^{***}p<0.001$$, $$^{**}p<0.01$$, $$^{*}p<0.05$$

**Table 4:** Point estimates for plot presented in Fig. 3 with percentage reduction in total crime and public transport passenger numbers observed between Feb. 29 to May 24, 2020, compared to counterfactual estimates based on ARIMA models estimated using data from Jan. 1, 2017 to Feb. 28, 2020

	Post-COVID-19 [95% CI]	Post-lockdown [95% CI]
All crimes	−30.6% [−43.4%, −14.1%]	−42.7% [−53.3%, −28.8%]
Violent robbery	−31.0% [−47.6%, −7.4%]	−42.7% [−56.6%, −22.7%]
Non-violent robbery	−30.9% [−51.2%, 1.4%]	−42.2% [−59.6%, −14.4%]
Robbery against residence	−45.6% [−67.1%, −3.4%]	−58.6% [−75.0%, −26.2%]
Serious violent crime (non-sexual)	−27.1% [−53.2%, 20.3%]	−39.0% [−61.0%, 1.1%]
Sexual violence	−53.3% [−78.4%, 21.5%]	−66.5% [−84.6%, −11.9%]
Domestic violence	−26.0% [−49.6%, 13.4%]	−36.5% [−57.1%, −1.9%]
VAW helpline calls	27.6% [−19.5%, 115.1%]	18.1% [−26.0%, 101.0%]
BRT + SCT passengers (in millions)	−53.3% [−70.1%, −22.7%]	−68.1% [−79.7%, −46.7%]

**Table 5:** Point estimates for plot presented in Fig. 4 for the estimated percentage change in daily crime rates for a 68.1% reduction in daily passenger numbers, using three time periods

	Complete period [95% CI]	Pre-COVID-19 [95% CI]	Post-lockdown [95% CI]
All crimes	−17.3% [−19.8%, −14.8%]	−16.5% [−19.1%, −13.9%]	−25.6% [−31.0%, −19.8%]
Violent robbery	−29.4% [−32.5%, -26.1%]	−26.6% [-29.7%, −23.4%]	−41.5% [−46.1%, −36.4%]
Non-violent robbery	−34.7% [−37.6%, -31.7%]	−33.1% [-36.5%, −29.6%]	−41.0% [−48.6%, −32.2%]
Robbery against residence	4.7% [−3.5%, 13.7%]	10.3% [2.3%, 18.9%]	10.2% [−42.3%, 110.5%]
Serious violent crime (non-sexual)	−8.8% [−15.6%, −1.4%]	−6.8% [−13.9%, 0.8%]	6.7% [−16.7%, 36.6%]
Sexual violence	−22.4% [−30.2%, −13.8%]	−18.7% [−27.2%, -9.2%]	−27.2% [−50.7%, 7.5%]
Domestic violence	0.0% [−5.8%, 6.3%]	2.3% [−3.8%, 8.8%]	12.2% [−12.7%, 44.2%]
VAW helpline calls	−14.4% [−20.4%, −7.8%]	−14.9% [−21.2%, −8.1%]	−1.9% [−33.8%, 45.4%]

## Footnotes

1 Statement made on Twitter: https://twitter.com/SSPCMexico/status/1288217671629180928.

2 See the website for Google’s COVID-19 Community Mobility Reports, https://www.google.com/covid19/mobility/ for more information. Accessed April 29, 2021.

3 Available at https://datos.cdmx.gob.mx.

4 The categories are: administration and finance, connectivity, COVID-19, economic development, culture, sport, social welfare, housing and development, education, environment and climate change, justice and security, infrastructure and public works, government accountability, citizen participation, mobility, tourism, and health.

5 Mexico City is technically a state.

6 As crime data are published monthly, the last week of May, 2020 was dropped to control for potential lags in reporting.

7 Robberies against residential dwellings with and without violence.

8 Violent crimes including homicide, assault, kidnapping, and extortion.

9 Includes rape and sexual assault.

10 Gender-related violence includes sexual and domestic violence against women.

11 To compare, public transport was the main means of transportation in only 35% of all London trips in 2017 (TfL, 2019).

12 The Hyndman-Khandakar ARIMA algorithm (Hyndman & Khandakar, 2008) replicates the steps an analyst would take to select the appropriate ARIMA order. In short, it first uses unit-root tests to determine whether differencing is needed to make the series stationary, then the values of *p* and *q* are determined using a stepwise search until the model with the smallest AICc is found (see also Hyndman & Athanasopoulos, 2019, ch. 9.7). Akaike’s information criterion (AIC) is a widely used model selection criterion used to compare models fit using maximum likelihood estimation, applying a penalty to the log-likelihood according to the number of parameters in the model. For a range of model specifications fit to the same data, the model with the smallest AIC represents the ‘optimal tradeoff between fidelity to the data and parsimony’ (Cavanaugh, 2007). However, the AIC has a small-sample bias that can lead to over-fitting, thus a corrected AIC (AICc) is generally used (Cavanaugh, 2007).

13 As the models with ARIMA errors estimated did not exhibit seasonal integration, only the non-seasonal case is described here.

14 When the coefficient is smaller than 1, the relationship is said to be inelastic, whereas when it is greater than 1 it is considered to be elastic.

15 An inspection of the VAW helpline calls residuals indicate that there was a cluster of significant lags at days 7 to 10, which could indicate a complex process of event dependence: for example, victims may be asked to call again in 10 days time for follow ups.

16 The greater uncertainty is expected given that the post-lockdown period contains considerably fewer observations.

17 Estimated deaths in Mexico City obtained from Mexico’s government excess mortality statistics: https://coronavirus.gob.mx/exceso-de-mortalidad-en-mexico/. Accessed May 31, 2021.
